# Facile chemical routes to mesoporous silver substrates for SERS analysis

**DOI:** 10.3762/bjnano.9.82

**Published:** 2018-03-14

**Authors:** Elina A Tastekova, Alexander Yu Polyakov, Anastasia E Goldt, Alexander V Sidorov, Alexandra A Oshmyanskaya, Irina V Sukhorukova, Dmitry V Shtansky, Wolgang Grünert, Anastasia V Grigorieva

**Affiliations:** 1 Lomonosov Moscow State University, Leninskie gory 1, bld. 73, Moscow, 119991, Russia; 2 Kurnakov Institute for General and Inorganic Chemistry of RAS, Leninsky prospect 31, Moscow 119991, Russia; 3 Skolkovo Institute of Science and Technology, Nobel str 3, Skolkovo, 143026, Russia; 4 National University of Science and Technology “MISiS”, Leninsky prospect 4, Moscow 119049, Russia; 5 Department of Chemistry and Biochemistry, Ruhr-University at Bochum, Universitätsstraße 150, Bochum, 44801, Germany

**Keywords:** meldonium, mesoporous silver substrates, silver oxide, surface-enhanced Raman spectroscopy

## Abstract

Mesoporous silver nanoparticles were easily synthesized through the bulk reduction of crystalline silver(I) oxide and used for the preparation of highly porous surface-enhanced Raman scattering (SERS)-active substrates. An analogous procedure was successfully performed for the production of mesoporous silver films by chemical reduction of oxidized silver films. The sponge-like silver blocks with high surface area and the in-situ-prepared mesoporous silver films are efficient as both analyte adsorbents and Raman signal enhancement mediators. The efficiency of silver reduction was characterized by X-ray diffraction and X-ray photoelectron spectroscopy. The developed substrates were applied for SERS detection of rhodamine 6G (enhancement factor of about 1–5 × 10^5^) and an anti-ischemic mildronate drug (meldonium; enhancement factor of ≈10^2^) that is known for its ability to increase the endurance performance of athletes.

## Introduction

Nowadays one of the largest sectors of the global chemical industry is the production of different mesoporous materials. Most of these materials are ordered or disordered oxides, which can be successfully produced by many methods such as wet chemistry [1–2], hydrothermal treatment [3], aerosol spray pyrolysis [4], etc. In contrast, metal mesoporous materials are rare due to the low redox potential of most of the metals, leading to fast oxidation or corrosion of metal sponges in ambient conditions. The oxidation stability of noble metals makes them more reliable as functional materials and thus materials such as porous silver, gold and platinum can be kept for a long time in air.

Mesoporous noble metals are mostly used as catalysts for high surface energy, gas sensor components, cell imaging mediators, etc. [5]. The most popular methods for mesoporous metal processing include acidic etching of bimetallic molts [6], electrochemical dealloying [7], electroplating using templates [8], electrophoretic deposition of nanoparticles [9] and diverse techniques of hollow porous structure formation, for example, by aerosol pyrolysis or by smart chemical etching. The latter includes selective chemical etching of one metal component [10–11] or etching during galvanic replacement [12]. Among other relevant strategies, nanoparticle aggregation [13–14] or direct deposition of porous films without templates [15–16] should be mentioned. Chemical etching is a technically low demand process – an important advantage of this technique. However, it requires that the leached metal be recycled. The etching process is the most efficient in the case of thin films or nanoparticles as no core or underlying layers remain unreacted. The concentration of the etching agent and duration of the treatment are the key kinetic factors to reach complete conversion of precursors [5].

Surface-enhanced Raman scattering (SERS) is a fast developing technique which originates from the theory of plasmonics and has already realized real applicable results important enough for industry [17]. Nowadays SERS spectroscopy is an easily accessible method for routine analysis (even using portable Raman spectrometers) or more complicated analytical tools [18]. Different kinds of dilute analytes can be easily identified in pristine form or in the form of tinted charge-transfer complexes as proposed by Sidorov et al. [19]. The analysis of analytes in the gas phase and the separate detection of tinted compounds in a mixture are more complex and still less efficient. Such analyses require specific SERS substrates with a high surface area, hierarchical surface structure and the presence of multiple plasmon bands (“polycolor” plasmons) in the visible range. For this, some prospective but technically complicated approaches were proposed elsewhere [20–22] while synthesis of mesoporous materials could be an alternative and easier solution [23]. Porous silver or gold materials of large specific surface area could be regarded as new metal plasmonic systems with complex architecture [24]. Pore distribution analysis is required for such materials as the first step towards a theoretical prediction of preferential adsorption and size selection factors.

Noble metals such as silver and gold are mostly employed as SERS spectroscopy platforms because of their pronounced surface plasmons with energy in the visible spectral range [25]. Silver is known to exhibit more intense plasmon oscillations compared to gold. Also, most of the elaborated synthesis techniques for mesoporous gold particles include different etching reagents hazardous for biological objects [6].

Recently Lyu et al. [26] proposed an efficient method for synthesis of silver polygonal structures with amazingly precise shape control using silver(I) oxide as a structural precursor. According to the technique described also elsewhere [27], polycrystalline uniform Ag_2_O polyhedron-like crystallites could be quantitatively obtained. Such cubic and octahedral crystallites are of interest as models for mesoporous silver synthesis.

In the present manuscript we used submicrometer-sized Ag_2_O cubes as precursors for mesoporous silver structures resulting from diffusion-limited reduction [26–27]. The same reduction method was also used to fabricate mesoporous silver films from partially oxidized sputtered silver films. Both mesoporous silver cubes and films act as effective SERS mediators as revealed by test experiments employing rhodamine 6G. We also demonstrated the detection of an anti-ischemic mildronate (meldonium) drug, which is widely known for its ability to increase the endurance performance of athletes, which resulted in its inclusion to the World Anti-Doping Agency (WADA) prohibited list.

## Results and Discussion

**Figure 1:**
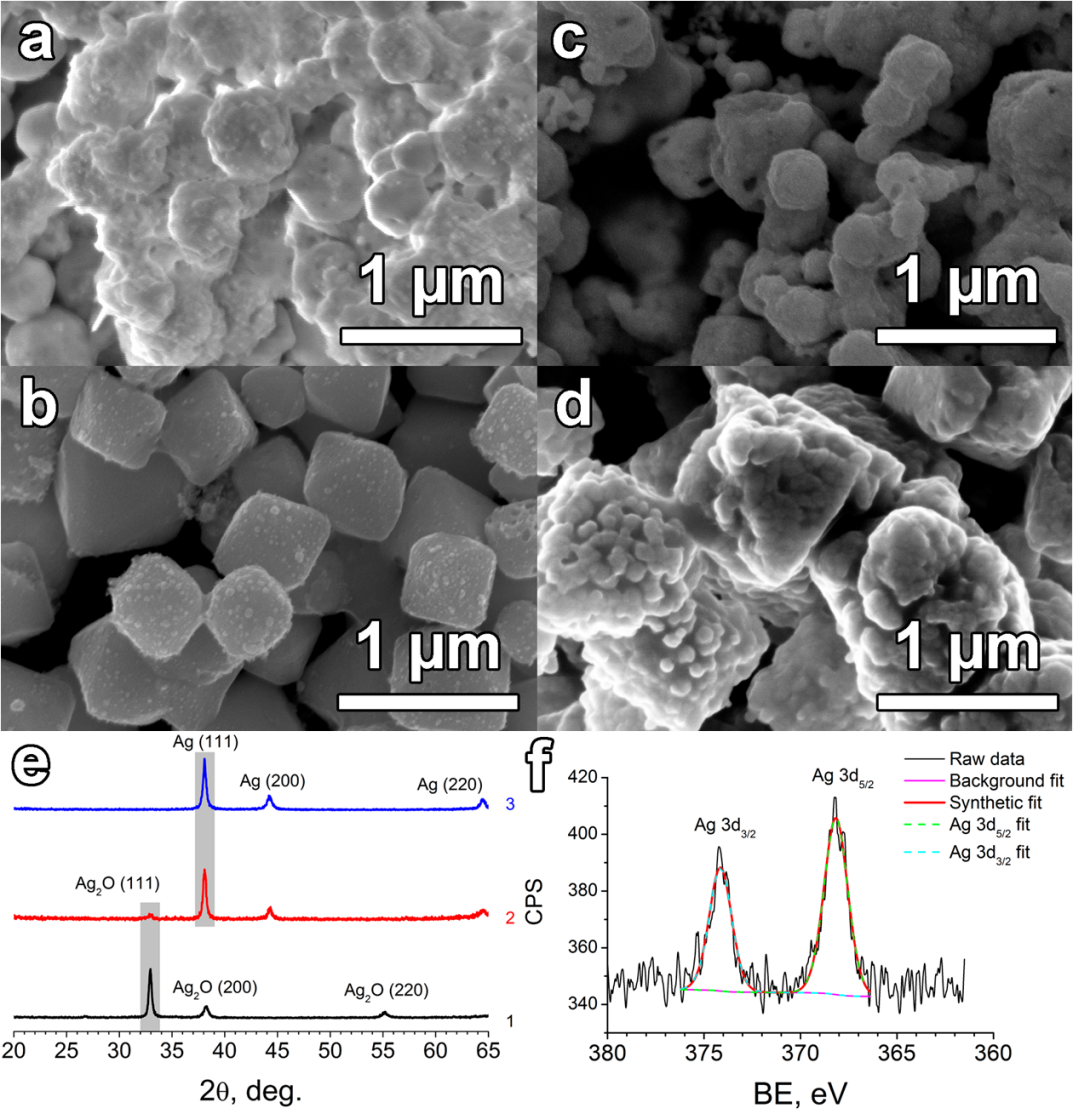
(a,b) SEM micrographs of Ag_2_O crystallites produced by alkaline sedimentation in excess of PVP, namely, (a) AgNO_3_/PVP 1:10 and (b) AgNO_3_/PVP 1:5. (c,d) SEM micrographs of the samples obtained by Ag_2_O reduction using NaBH_4_ with the following molar ratios: (c) Ag_2_O/NaBH_4_ 1:2 and (d) Ag_2_O/NaBH_4_ 1:10. The reducing treatment time was 40 min for both the samples. (e) XRD data for pristine Ag_2_O (1) and reduction products at double excess of NaBH_4_ (2) and ten-fold excess of NaBH_4_ (3). (f) XPS spectra of silver mesoporous particles (mp-Ag) obtained in a Ag_2_O/NaBH_4_ 1:10 ratio.

Following the procedure proposed by Lyu et al. [26], Ag_2_O cubic crystallites were synthesized by alkaline precipitation from a 0.1 M silver nitrate solution in the presence of poly(vinyl pyrrolidone) (PVP, *M*_w_ ≈40000 kDa). The Ag/PVP molar ratio was varied to optimize the phase composition and micromorphology of the product. The microstructure of the products varied with the molar ratio of the reactants (Figure 1a,b). A roughly equimolar ratio of silver and PVP (monomer units) leads to randomly shaped and highly merged oxide particles, while a ten-fold excess of polyol produced well-developed cube-like crystallites with the mean size of 480 ± 70 nm. Obviously, during the oxidation stage, PVP molecules play an important role as surfactant, partly blocking the nucleation at (100) facets if the prevalent growth mechanism is normal or lateral layer-by-layer growth [28]. Presumably, the PVP adsorbates remained at the Ag_2_O crystallite surface after the washing procedure. Remarkably, the porous polyhedron-like silver structures could be also synthesized using aerosol spray pyrolysis as described in [29–30].

The following reduction of Ag_2_O was performed using three different concentrations of NaBH_4_ solution. A 100-fold excess of NaBH_4_ led to instantaneous decomposition of Ag_2_O particles, producing a black-colored colloid of metal silver. The double excess of reducing agent showed a less efficient reduction process than the ten-fold excess. The corresponding XRD graphs related to the reduction products after 40 min are given in Figure 1e. At a double molar excess of NaBH_4_ the (111) reflection of silver(I) oxide decreased remarkably while the Ag_2_O (200) reflection vanished completely. At the same time, broadened metal silver (111), (222) and (220) peaks appeared. According to XRD data, a ten-fold excess of NaBH_4_ led to the complete reduction of cube-like Ag_2_O crystallites.

The mean particle size of mesoporous silver prepared at a double excess of NaBH_4_ is of 230 ± 90 nm (Figure 1c) and the related pore size is about 100 nm. A ten-fold excess of NaBH_4_ led to full conversion of polycrystalline Ag_2_O to ≈1 μm sponge-like Ag grains (Figure 1d), which also contained numerous mesopores. As it was discussed elsewhere [12], the pore formation arises from the (100) facet etching in the same way as it was observed previously for polygonal silver structures [26].

This is noticeable in the case of ten-fold NaBH_4_ excess, where the characteristic grain dimensions exceed the size of pristine Ag_2_O cubic crystallites. Most likely, this effect is related to recrystallization processes, which include removing silver ions from the lattice and its further deposition onto the exterior surface of the domains when reduced. Such a diffusion-limited reduction is the processing pathway towards a porous material with uniform pores.

According to the Brunauer–Emmett–Teller (BET) surface area analysis and the Barrett–Joyner–Halenda (BJH) (BET-BJH) nitrogen capillary adsorption analysis, the specific surface area of the mesoporous silver particles (mp-Ag) reached 42 ± 5 m^2^/g and the pore size distribution analysis demonstrated a broad maximum at 10–60 nm. The achieved value of the specific surface is rather high in comparison to similar materials based on metal silver [13]. The characteristic nitrogen adsorption–desorption isotherms are given on Figure S2, Supporting Information File 1.

The XPS data were applied for analysis of the valence state of the silver at the surface of the mp-Ag (Figure 1f) obtained in 1:10 Ag_2_O/NaBH_4_ molar ratio. The binding energies at 368.3(2) eV and 374.2(2) eV are related to Ag 3d_5/2_ and Ag 3d_3/2_ binding energies, respectively. According to the NIST database (CAS registry No 7440-22-4) these bands correspond to the metal state of silver [31]. Both characteristic energies are decreased slightly, probably, as a result of PVP adsorbates at the surface. The absence of a silver oxide phase at the surface is also beneficial for efficient surface plasmon resonance, which is strongly required for SERS. This is also in concordance with XRD analysis, which revealed no reflections of crystalline Ag_2_O after reduction by ten-fold excess of NaBH_4_ (Figure 1e).

**Figure 2:**
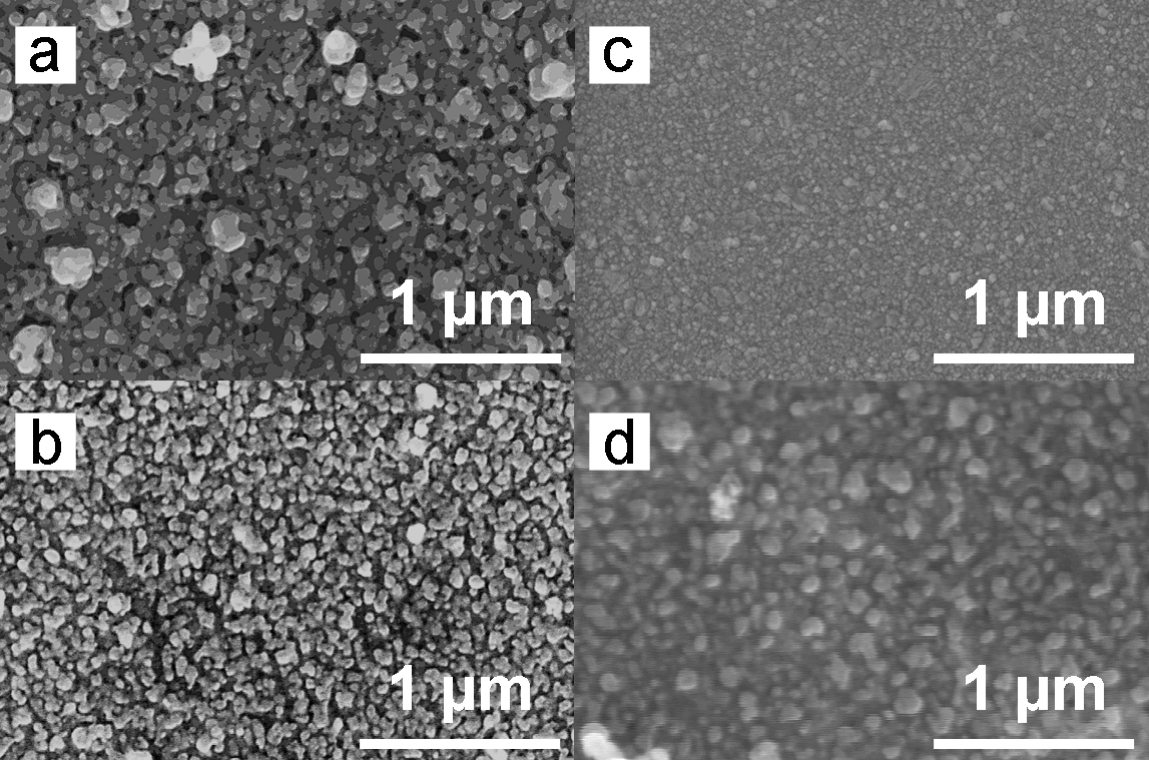
SEM images of mesoporous silver films after immersion for 40 min in NaBH_4_ (a) without PVP and (b) with 5 mM of PVP, respectively. In (c) and (d) the micrographs of initial magnetron silver film and Ag_2_O films are presented.

The similar chemical reduction procedure was applied then for mesoporous Ag film formation. The primary silver film with an estimated thickness of 150 nm was deposited onto the 2.5 × 2.5 cm glass slides using magnetron sputtering. The chemical oxidation of silver was performed by vapors of 63% nitric acid. Then, the partially oxidized film was chemically reduced in an excess of sodium borohydride to Ag^I^ in the presence of or without PVP. The concentration of PVP in the aqueous NaBH_4_ solution was 5 × 10^−3^ M per monomer. The tuning of the silver(I) oxide behavior was not actualized by variation of the reductant concentration, because low concentrations of NaBH_4_ required longer immersion of the film, which increased the risk of delamination. Namely, for the films immersed to the aqueous medium for ≈60 min, strong delamination was observed. Empirically, 40 min reduction was found to be optimal to achieve effective Ag_2_O reduction with minimal film delamination. The reduction of Ag_2_O films was carried out in individual NaBH_4_ solutions and, alternatively, by using NaBH_4_ mixed with PVP (5 mM PVP concentration). The microstructure of the resulting films was examined by SEM (Figure 2). The XRD data for the reduced films showed only broadened metal silver reflections. For an example, see Figure S3 in Supporting Information File 1.

In both the cases the reduction of Ag^I^ was efficient, leading to mp-Ag/Ag porous films, however the microstructure was different. Both films were mesoporous with an individual grain size in the range of 20–100 nm in length. The average particle diameter for mp-Ag/Ag was 48 ± 12 nm for the sample reduced without PVP, while in presence of PVP, the average particle diameter was 33 ± 10 nm. The characteristic feature of the crystallites was sphere-like or ellipse shaped. PVP produced no macroporous aggregates, because no large single crystals of Ag_2_O were in the film, but improved the porosity of the Ag film (Figure 2b). Likely, PVP molecules adsorb at growing Ag particles, controlling their growth and leading to the spherical shape.

**Figure 3:**
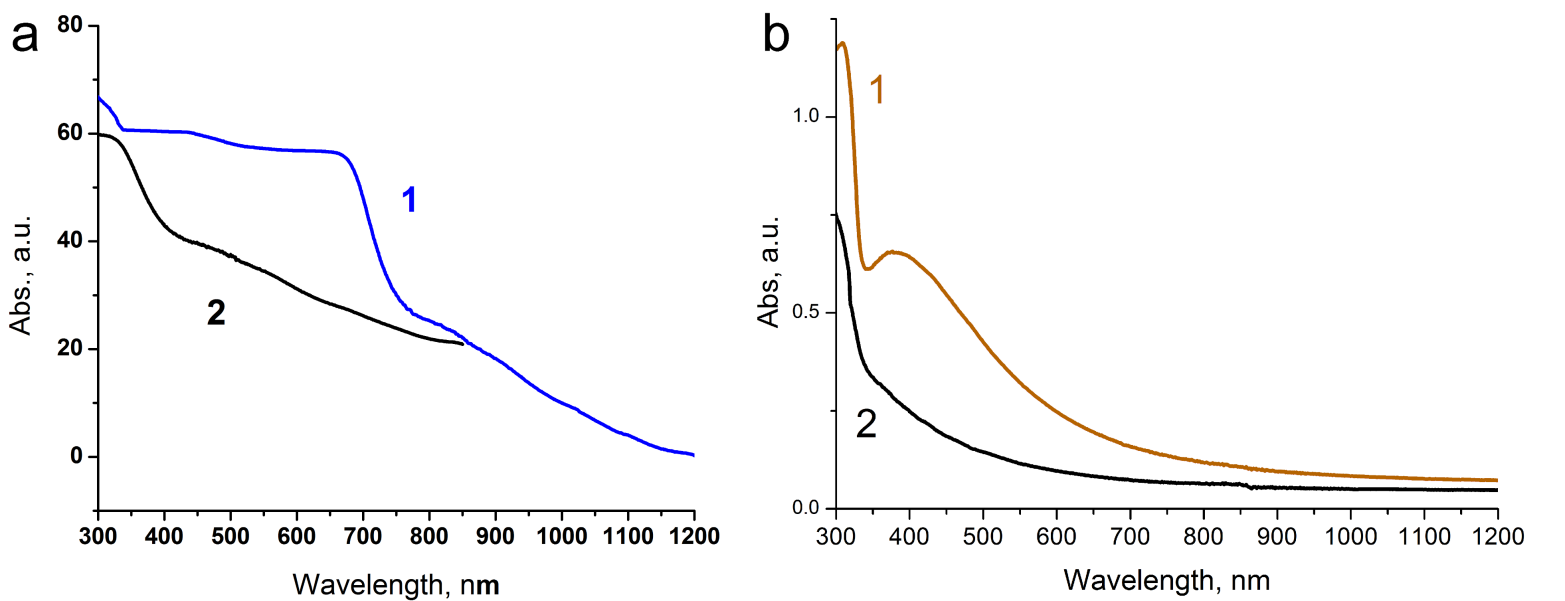
Optical absorbance spectra replotted from total reflectance data collected by an integrating sphere (*d* = 150 mm): (a) (1) reduced by NaBH_4_ silver and (2) pristine Ag_2_O polycrystals, and (b) (1) mp-Ag/Ag (PVP) film, (2) pristine Ag_2_O film.

The mp-Ag/Ag slides were yellow-tinted because of surface plasmon resonance effect, which was more pronounced for films with smaller grain size. For the PVP-assisted film, the optical absorbance spectrum, replotted from total reflection data, is presented in Figure 3.

The water contact angle was measured for the reduced and initial Ag_2_O films. The contact angle for water aliquots of 0.5 µL was of 20 ± 5° for six Ag_2_O films obtained using standard methods with a net 150 nm thick silver film oxidation in vapors of concentric nitric acid. The average contact angle value for reduced films varied depending on the roughness of the film while the presence of PVP molecules in the reaction media was less significantly influenced. For uniform nanostructured reduced films, the contact angle increased up to 59 ± 11° in both cases. For comparison, the contact angle value for defective (with small scratches) films were 52 ± 15°.

The mesoporous silver microcubes and mp-Ag/Ag films prepared employing PVP and a ten-fold excess of NaBH_4_ were compared as promising materials for SERS chips.

The Raman spectroscopy of rhodamine 6G (R6G) was performed using 5 μL droplets and the concentration range of the analyte was 10^−8^–10^−6^ M. The corresponding Raman spectra for R6G deposited onto pristine silver oxide samples contained no signal from the model analyte but only noisy background, indicating luminescence processes from the dye.

**Figure 4:**
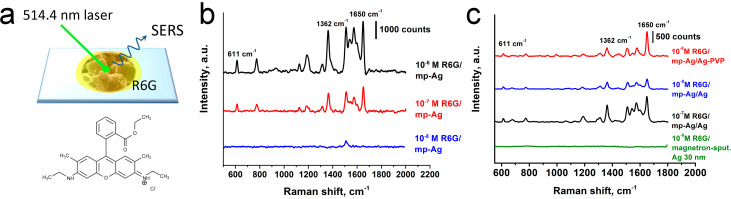
(a) The principal scheme of SERS experiment and (b) corresponding SERS spectra of rhodamine 6G in 10^−8^ M, 10^−7^ M and 10^−6^ M concentrations obtained using the substrates of mesoporous Ag microcubes synthesized at Ag_2_O/NaBH_4_ 1:10 ratio. (c) SERS spectra of rhodamine 6G in 10^−7^ M and 10^−8^ M 1–2 μL aliquots collected using mp-Ag/Ag slides prepared with or without PVP.

It was surprising that the mp-Ag deposited onto a glass slide and the mp-Ag/Ag slides of porous silver demonstrated a detection limit for the model dye of 10^−8^–10^−9^ M, which is close to the characteristics for most thick porous substrates [32]. However, the spectra for the 10^−9^ and 10^−8^ M concentration are rather poor and only contained the known weak C–C stretching modes at 1508 cm^−1^ (Figure 4b), while all other peaks of R6G vanished or broadened to form a significant background contribution. Feasibly, the effect of a noisy background is due to the diverse types of pores in the materials when the derived surface serves as a source of surpassing scattering.

The typical SERS spectra of R6G for 10^−7^ M and 10^−6^ M analyte concentrations show intensive stretching vibrations of carbon aromatic skeleton of the molecule at 1362(s), 1508(s), 1580(m), 1601(s), and 1650 (s) (Figure 4b) [33]. The enhancement factor (EF) for the system was calculated as described according to the standard equation, namely,


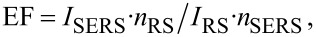


where *I*_SERS_ and *I*_RS_ are the corresponding SERS and Raman spectroscopy signal intensities and *n*_SERS_ and *n*_RS_ are the molar quantities of R6G in SERS and Raman experiments. As the most appropriate peak for analysis of the EF, 1362 cm^−1^ was taken as the sharpest and the most evident with a combination of the background registered for the given concentrations of the analyte. The EF was found to be ≈1–5 × 10^5^ for 10^−7^ and 10^−6^ M concentrations, while for lower concentrations, it changed drastically. These values are lower than the EF of 10^9^ reported for 10^−9^–10^−12^ M aliquots of crystalline violet deposited on mesoporous silver mesocrystals enhanced with a 633 nm He–Ne laser [23].

Concerning the mp-Ag/Ag slides, the corresponding SERS spectra of R6G (Figure 4c) showed a rather intense signal of the dye already at 10^−8^ M. The most intense SERS bands in the spectra were related to ν(C–C) stretching vibrations of the aromatic core and, namely, 1362(s), 1508(s), 1540(m), 1573(s), 1602(w), and 1650(s) cm^−1^ were observed to identify R6G. The bands in the lower Raman shift range were 1310(m) cm^−1^ (C–O–C stretching) and 1184(w), 1126(s), 990(w) cm^−1^, 777(m) cm^−1^ and 611(m) cm^−1^ corresponded to bending C–H vibrations in R6G [34]. These sharp peaks were less intensive in the spectra of R6G deposited onto mesoporous Ag blocks. Most likely, this results from better wettability of porous Ag blocks. Probably, the presence of macropores in the porous Ag aggregates leads to better penetration of the dye into the pores, decreasing the effective percentage of the analyte at the surface. The mp-Ag/Ag slides demonstrated worse wettability and better uniformity at the surface, which is much more appropriate for routine SERS analysis of various water-based compounds.

**Figure 5:**
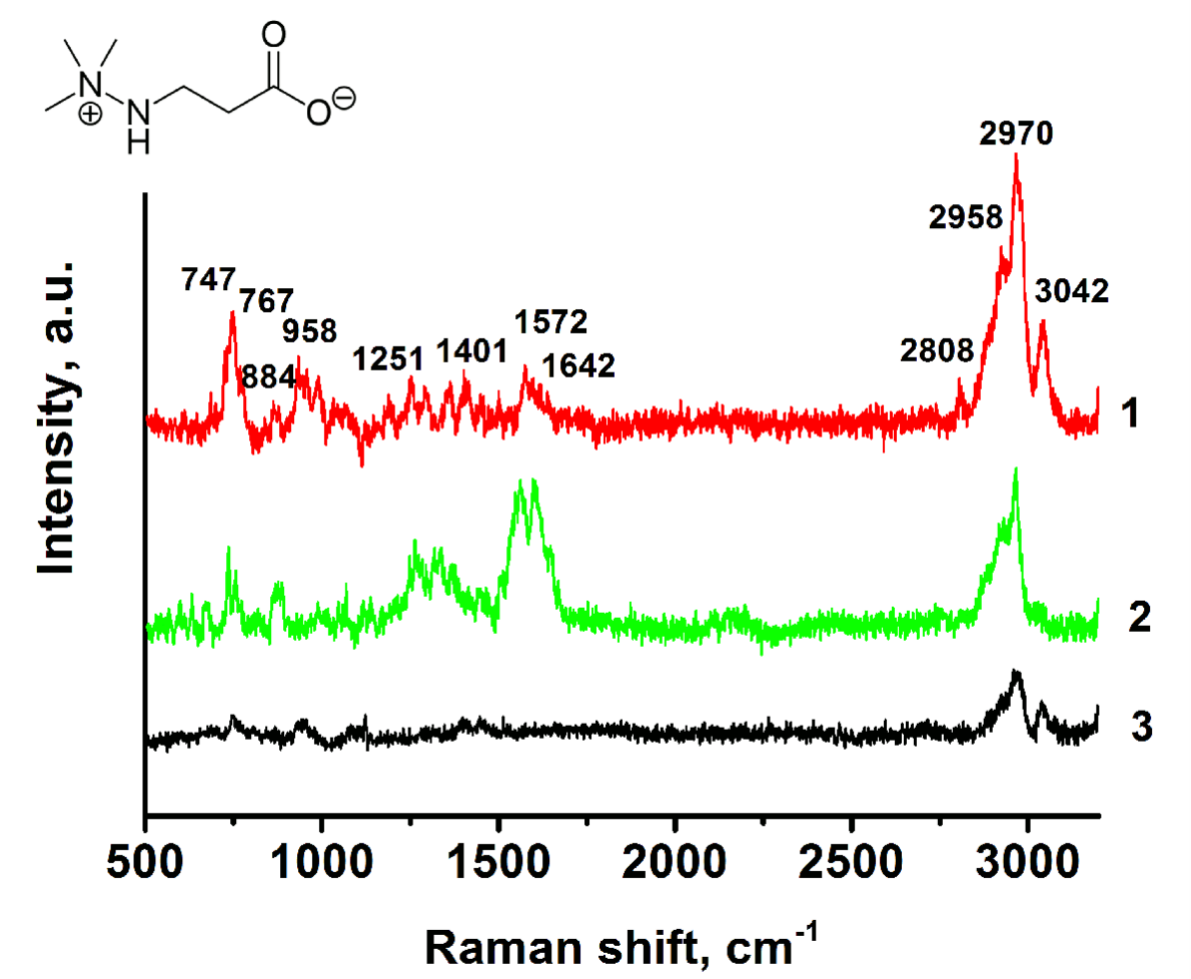
SERS spectra of mildronate in (1) 10^−3^ M and (2) 10^−2^ M concentrations collected using mp-Ag/Ag slides prepared in the presence of PVP. The volume of the aliquot was 2 μL. As a reference sample, 2 µL of 100 mg/mL standard solution was dried on the glass substrate (3). The excitation laser was 514.4 nm, at 5% power of 20 mW, and 10 s acquisition time.

As a key analyte in current manuscript, meldonium (mildronate) was applied to demonstrate SERS activity of the substrates using tintless compounds. Meldonuim is an anti-ischemic drug and a metabolic modulator that was added to the World Anti-Doping Agency’s (WADA) prohibited list as a doping substance [35]. The corresponding analyte was investigated as an aqueous solution of 1 × 10^−3^ M and 1 × 10^−2^ M total concentrations. We used its unique Raman spectra from the source [36] to identify the substance. The most intense spectral bands in the spectra there are 747(s), 767(m), 861(m), 884(m), 934(m), 984(m), 1086(m), 1251(m), 1293(m), 1358(m), 1408(m), 1449(m), 1572(s), 1600(m), 1642(m), 2808(m), 2958(s), 2970(s), and 3042(s) cm^−1^ bands. A number of Raman modes observed in Figure 5 could be associated with characteristic Raman modes for trimethylhydrazine, which has similar groups in its structure. The similar vibrations are 747(s), 884(m) (skeletal stretching), 1086(m) (CH_3_ rocking), 1408(m) (CH_3_ bending), 1449(m) (CH_3_ bending), 1572(s) and 1600(m) (NH_2_ bending), 2958(s) and 2970(s) (CH_3_ stretching), 3042(s) (CH_3_ stretching) [37]. The survey Raman spectrum of meldonium was registered as a reference sample. The overall profile of the spectrum is similar to that presented in the literature [36].

The EF value for meldonium was estimated using the characteristic 747 cm^−1^ C–C stretching mode and 2970 cm^−1^ of CH_3_ stretching band for the calculations. The EF value for the 10^−3^ M probe was 1.5 × 10^2^ for the 747 cm^−1^ mode and 0.9 × 10^2^ for the 2970 cm^−1^ mode.

**Figure 6:**
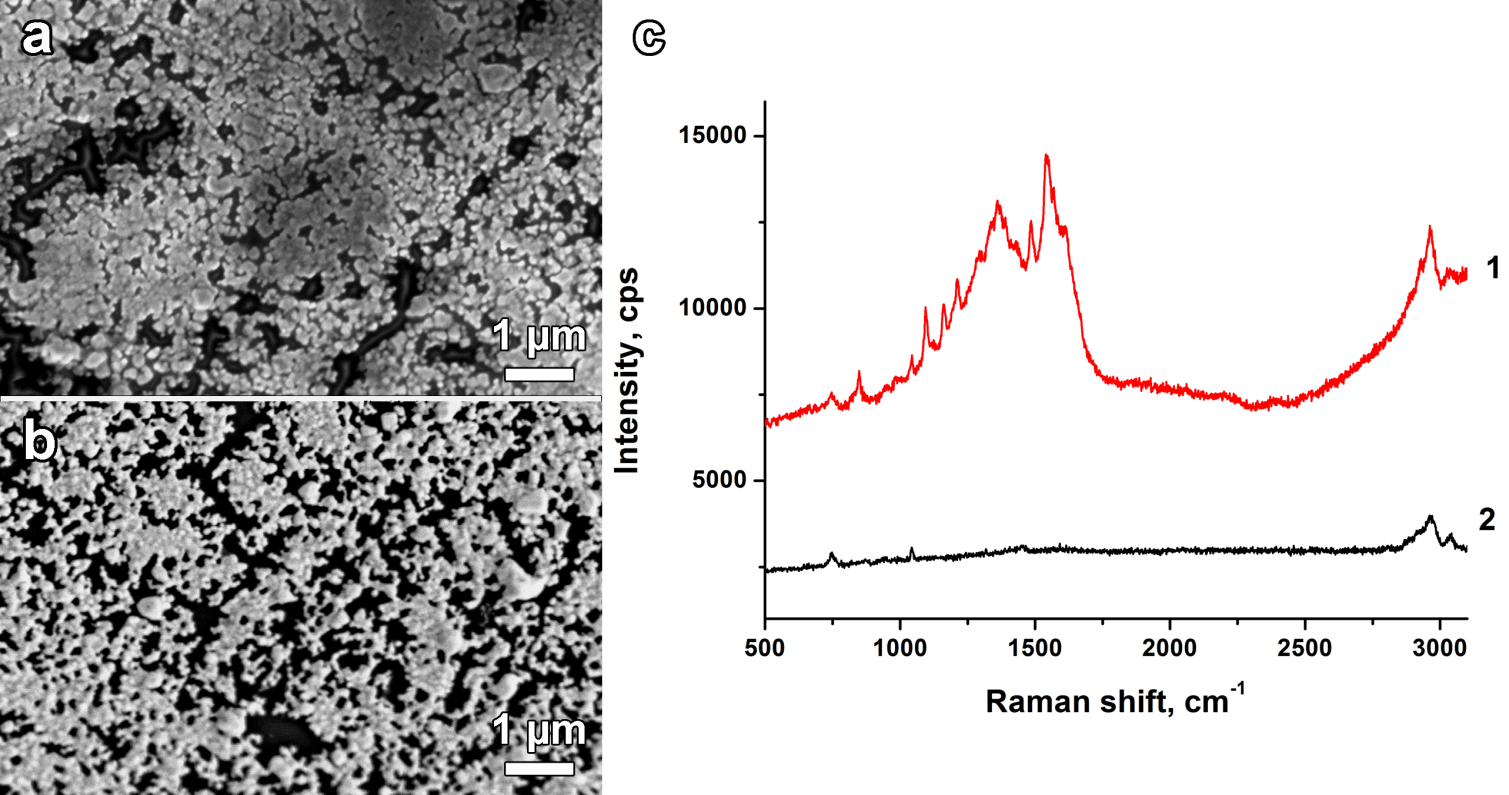
Micrographs of mp-Ag/Ag slides prepared in the presence of PVP after sintering for 1 h in air at (a) 110 °C and (b) 120 °C. (c) Raman/SERS spectra of meldonium (probe: dried 2 μL probe of 1 × 10^−2^ M concentration) after sintering at (1) 110 °C and (2) 120 °C for 1 h in air. The excitation laser wavelength was 514.4 nm, at 5% power of 20 mW, and 10 s acquisition time.

The microstructure of the nanostructured films applied as SERS chips in SERS experiments above showed advanced thermal stability up to 110 °C. The films were sintered at 110 °C and 120 °C for 1 h in air to remove any condensed compounds adsorbed at the surface after SERS experiments. Figure 6 shows micrographs of the mp-Ag/Ag films after calcination in air at 110 °C and 120 °C for 1 h, respectively. The corresponding micrographs show insignificant changes in microstructure after 110 °C sintering while fresh crack formation was revealed. In Figure 6b the film is destroyed completely, and Ag particles hardly cover half of the glass slide surface. Such low temperature melting of nanocrystalline silver is typical and was also observed for different forms of highly disperse silver and other noble metals.

Thermal stability is an important criterion for porous compounds in gas phase processes because of their enhanced adsorption behaviour and chromatography function. Analysis of the thermal stability showed low microstructural changes upon sintering at 110 °C while sintering at 120 °C degraded the material. We therefore recommend 100 °C as the maximal temperature for thermal stability of such a fine microstructure. The analysis of the SERS activity of sintered mp-Ag/Ag films was performed for meldoninum which was deposited before the calcination processing. It was found that after calcination at 110 °C, the SERS spectra of the bioanalyte are still observed. In contrast, the Raman spectra detected on substrates after 120 °C sintering showed rather poor and weak signal (Figure 6c). Such an observation is not due to meldonium evaporation but is due to the nanostructured silver recrystallization and degradation processes.

## Conclusion

In summary, an efficient and convenient synthesis method was proposed for mesoporous silver micrometer and submicrometer-sized particles. The obtained porous silver cubes were found to be highly sensitive SERS substrates with an enhancement factor of 10^5^. It was also evident that the mesoporous silver particles are of great functionality and could likely be promising not only as SERS substrates for liquid analysis, but also for gas phase SERS analysis and as catalysts in combustion and mild selective oxidation processes.

This same special chemical reduction method led to the easy scaling of uniform mesoporous silver films. These uniform mesoporous silver films served as SERS substrates and demonstrated a detection limit below 10^−8^ M for the same standard probe of R6G. The nanostructured silver films, obtained by an easy chemical route, are also promising for qualitative chemical analysis. In this work, very dilute pharmaceuticals, such as meldonium, could be detected using several microliters of probe material using the SERS spectroscopy technique.

## Experimental

The synthesis of silver(I) oxide nanocubes was performed using a procedure reported by Lyu et al. [26]. Briefly, 0.05 g of crystalline silver nitrate (Carl Roth GmbH, ≥99%, Ph.Eur., extra pure) was dissolved in 210 mL of 0.2 M ammonium nitrate NH_4_NO_3_ aqueous solution. PVP solution was added slowly in the PVP monomeric unit/silver at atomic ratios of 5:1 or 10:1. The corresponding concentration of PVP was about 5 × 10^−3^ M and 1 × 10^−2^ M. Then an excess of sodium hydroxide NaOH (450 mL of 0.2 M solution) was added and the reaction mixture was kept in the dark for 1 h under stirring. The overall molar ratios of the compounds were the following AgNO_3_/NH_4_NO_3_/NaOH 1:4.2:8.5. After multiple centrifugation steps of the precipitate and washing with ≈1 L of MilliQ pure water, the product was ready for the following syntheses. The dry product, which is appropriate for storage and further dispersion in water, could be produced freeze drying.

All the Ag particle synthesis was performed in a glass dish preliminary rinsed with 2 M nitric acid and then by an excess of distilled water to remove all possible reductants and dust. The reductant, namely, sodium borohydride (Aldrich, granular ≥98%), was dissolved in precooled 4 °C MilliQ water right before the experiment. For each experiment, 10 mL of 0.1 M presonicated aqueous suspension of Ag_2_O nanocubes was reduced using dropwise addition of the reductant solution. The reduction reactions were performed with double and ten-fold excess of NaBH_4_ to Ag^+^ ions. The reduction process was performed for 15 or 40 min and then the dark-colored product was washed out and dried in air at ambient conditions.

A mesoporous Ag film was prepared in two steps using a magnetron sputtered silver film (thickness of 150 nm) as a precursor. The primary Ag film was deposited onto a thoroughly washed glass slide in argon using a magnetron Quorum Technologies Q150T turbo-pumped sputter coater/carbon coater. A standard silver disk sputtering target (Stanford Materials, 99.99%) served as a source and the deposition rate was 2 nm/s.

Then, the silver film was oxidized in the vapors of concentrated nitric acid (Reachim, purus). The silver film was fixed face down in 5 cm over 10 mL of acid in a glass vessel. The film was treated for 10 min in vapor and then thoroughly washed with MilliQ pure water. The film, which changed in color, was then immersed into 100 mL of freshly prepared 0.1 M NaBH_4_ aqueous solutions of (the same concentration as was taken for mesoporous polyhydrons when taken in ten-fold excess). The effect of immersion time was tested in 15–60 min durations of the process. To reveal the role of PVP on the morphology of the metallic silver, one of the reductant solutions also contained 5 × 10^−3^ M of PVP. The resulting plates were washed with distilled water and dried in ambient conditions for 24 h.

Scanning electron microscopy (SEM) was applied to characterize microstructure of the individual Ag or Ag_2_O nanostructures and films. The analysis was performed in using a NVision 40 microscope (Carl Zeiss) at 9 kV accelerating voltage. Statistical analysis of the micrographs was performed using ImageJ software.

The X-ray diffractograms were collected using the diffractometer RIGAKU D/max-RC with a 12 kW beam gun and a rotating copper anode (Cu Kα radiation, θ−2θ Bragg−Brentano geometry, 20−60° 2θ range, 0.02° step). The phase analysis of the compounds was carried out using WinXPow software and the ICDD PDF2 database.

X-ray photoelectron spectroscopy analysis was performed using an electron spectroscopy for chemical analysis (ESCA) setup at the Laboratory of Industrial Chemistry of Ruhr University at Bochum equipped with the X-ray source Specs XRC 1000, UHV chamber up to 10^−6^ mbar, and energy analyzer power PS-EA10N. For the sample preparation, a droplet of the colloid, preconcentrated by centrifugation, was deposited onto the carbon substrate and then dried in ambient air. To additionally concentrate the sample, ten droplets of the colloid were deposited and dried one by one. The specific surface area of the samples was determined by low-temperature nitrogen adsorption with an ATX-06 analyzer (KATAKON) by the five-point Brunauer–Emmett–Teller (BET) method.

The optical absorbance spectra of the films were collected using a Perkin Elmer Lambda 950 UV/Vis/NIR spectrophotometer in total reflectance regime. The samples were placed onto the reflectance port of a Spectralon-coated integrating sphere (*d* = 150 mm). The specular and diffusive reflectance signals were detected in the 300–1200 nm range (1 nm step) and replotted in absorbance coordinates.

All SERS spectra were collected using an InVia Reflex Raman confocal microscope (Renishaw Inc.) equipped with a 20 mW 514.4 nm argon laser and power neutral density filter (10%) with 60 s acquisition time. All spectra were collected using a confocal microscope Leica DMLM (resolution up to 2.5 μm) with a 50× objective lens. The diffraction grating was 2400 lines/mm, and the CCD camera had 1024 × 256 pixels. A standard (100) single crystalline silicon substrate was used for calibration. The analyte droplets (about 1–2 μL) were placed on the substrate and measured within ten minutes after deposition.

## Supporting Information

Experimental details.

File 1 Details:
1. Micrographs of the products obtained in different reagent ratios.
2. Micrographs of the reduction products obtained using H_2_O_2_ and N_2_H_6_SO_4_ instead of NaBH_4_.
3. Nitrogen adsorption plot for the Ag mesoporous nanocube sample synthesized with the molar ratio of 1:4 Ag_2_O/NaBH_4_.
4. XRD data for a bare Ag_2_O film and mesoporous Ag film synthesized with the molar ratio of 1:10 Ag_2_O/NaBH_4_ in 15 min reduction processing.
5. Experimental details on the wettability contact angle measurements.
6. XPS survey spectra for mesoporous aggregates.
