# Supplementary material for: Facile chemical routes to mesoporous silver substrates for SERS analysis
Source: Beilstein J Nanotechnol. 2018 Mar 14;9:880–9. doi: 10.3762/bjnano.9.82 (PMC5870146; doi:10.3762/bjnano.9.82)
Supplement: File 1 — Details: 1. Micrographs of the products obtained in different reagent ratios. 2. Micrographs of the reduction products obtained using H2O2 and N2H6SO4 instead of NaBH4. 3. Nitrogen adsorption plot for the Ag mesoporous nanocube sample synthesized with the molar ratio of 1:4 Ag2O/NaBH4. 4. XRD data for a bare Ag2O film and mesoporous Ag film synthesized with the molar ratio of 1:10 Ag2O/NaBH4 in 15 min reduction processing. 5. Experimental details on the wettability contact angle measurements. 6. XPS survey spectra for mesoporous aggregates. [file Beilstein_J_Nanotechnol-09-880-s001.pdf]

Supporting Information

for

Facile chemical routes to mesoporous silver substrates for SERS analysis

Elina A. Tastekova[1], Alexander Yu. Polyakov[1], Anastasia E. Goldt[1,2,3], Alexander V. Sidorov[1], Alexandra A. Oshmyanskaya[1], Irina V. Sukhorukova[4], Dmitry V. Shtansky[4], Wolfgang Grünert[5] and Anastasia V. Grigorieva[*1,§]

Address: [1]Lomonosov Moscow State University, Leninskie gory 1, bld. 73, Moscow, 119991, Russia, [2]Kurnakov Institute for General and Inorganic Chemistry of RAS, Leninsky prospect 31, Moscow 119991, Russia, [3]Skolkovo Institute of Science and Technology, Nobel str 3, Skolkovo, 143026, Russia, [4]National University of Science and Technology "MISiS", Leninsky prospect 4, Moscow 119049, Russia and [5]Department of Chemistry and Biochemistry, Ruhr-University at Bochum, Universitätsstraße 150, Bochum, 44801, Germany

Email: Anastasia V. Grigorieva - anastasia@inorg.chem.msu.ru

* Corresponding author

[§] Fax.: +7 (495) 939 09 98

Experimental details

Synthesis of silver (I) oxide nanocubes was performed using a procedure reported by Lyu et al. [1]. Briefly, 0.05 g of crystalline silver nitrate (Carl Roth GmbH, ≥99% , Ph.Eur., extra pure) have been dissolved in 210 mL of 0.2 M ammonium nitrate NH_4NO_3 aqueous solution. PVP solution was added slowly in a ratio 2:1 (PVP monomeric unit / silver atom). The corresponding concentrations of PVP were of about 5×10^{-3} M. Next an excess of sodium hydroxide NaOH (450 mL of 0.2 M solution) was added at the first onset and then preserved in dark for 1 h under stirring. The overall molar ratios of the compounds were as the following 1 $AgNO_3$: 4.2 NH_4NO_3 : 8.5 NaOH. Varying the NH_4NO_3 concentration in the range of 2×10^{-3} – 8×10^{-3} M hardly influenced on the resulting micromorphology of the product (Figure S1-a,b). After multiple centrifugations of the precipitate and washing by ~1 liter of MilliQ pure water the product was ready for the following syntheses. The dry product could be estimated using freeze drying method that is appropriate for storage and further dispersion in water.

All the Ag particle syntheses have been performed in glass dish preliminary rinsed with 2 M nitric acid and then by an excess of distilled water to remove all possible reductants and dust. The reductants, namely, sodium borohydride (Aldrich, granular ≥98%), hydrogen peroxide (Reachim, ≥33%) and hydrazine sulfate (Reachim, granular, purum) have been kept as solids and dissolved in preliminary cooled to 4 °C aqueous media (MilliQ water) right before the experiment. Hydrogen peroxide was taken after IREA200 as a purum reagent of 37 wt % saturated aqueous solution. For each experiment 10 mL of 0.1 M preliminary sonicated aqueous suspension of Ag_2O nanocubes was reduced using dropwise addition of the reductants. The reduction was performed with a double excess of each reducing agent, namely H_2O_2, $N_2H_6SO_4$ or $NaBH_4$, to Ag^+ ions. Microstructure of the samples are given in Figure 1c,d and Figure S1c,d. Diluted solution of hydrogen peroxide demonstrated low efficiency in Ag_2O reduction producing incomplete reduction. In contrast, hydrazine sulfate produced highly porous silver structure which had nothing common with initial micromorphology Ag_2O polyhydron crystallites. In the issue, as a reductant the ten-fold excess of $NaBH_4$ to Ag_2O was applied as described above. This reagent is effective but less destructive for the primary microstructure than the hydrazine sulfate.

Reduction with a ten-fold excess of $NaBH_4$ was also carried out leading to mesoporous structures with less degradation of secondary structure of the polyhedrons. The reduction processing was performed for 15 or 40 min and then the dark-colored product was washed out and dried in air at ambient conditions. The micrographs of the corresponding products are given in Figure 1.

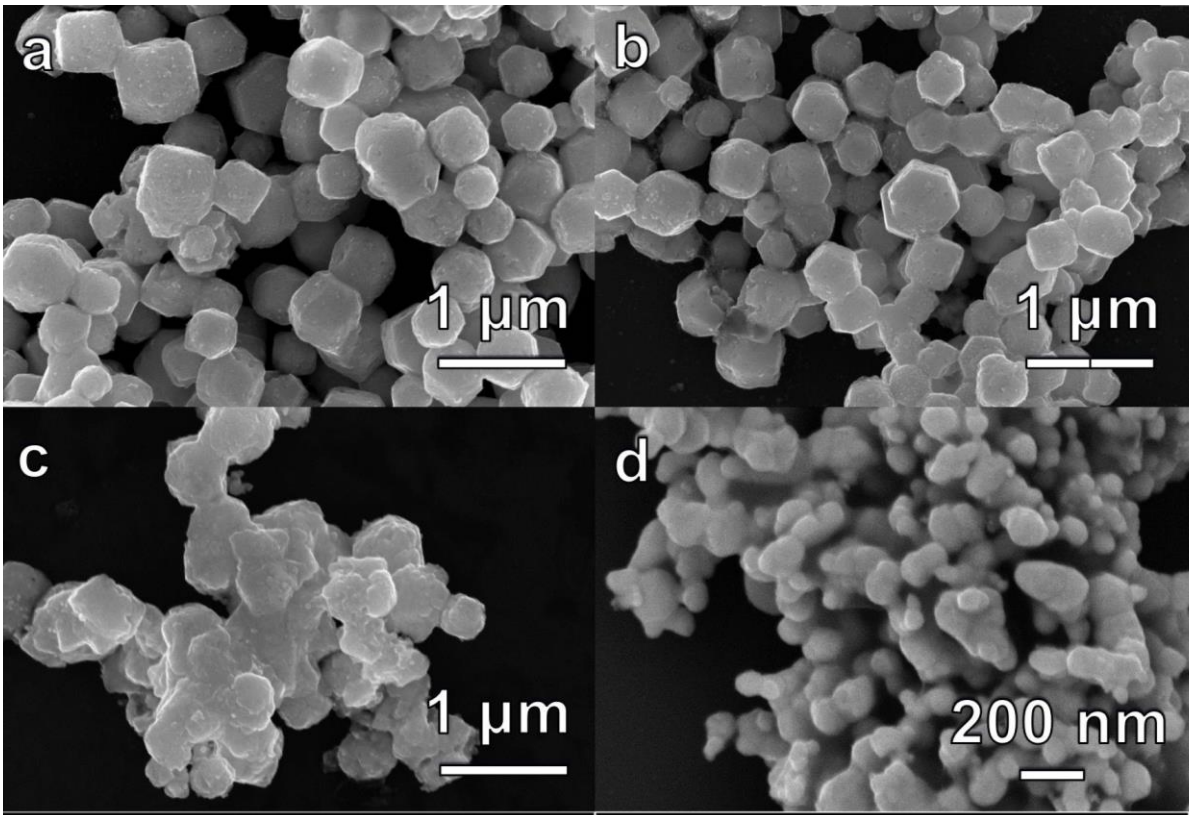

Figure S1: SEM micrographs of silver oxide and mesoporous silver samples prepared using different ratios of silver nitrate/ammonium nitrate/sodium hydroxide: (a) Ag_2O sedimented in 1 $AgNO_3$: 4.2 NH_4NO_3 : 8.5 NaOH reagent ratio, (b) (a) Ag_2O sedimented in 1 $AgNO_3$: 8 NH_4NO_3 : 8.5 NaOH reagent ratio , (c) reduction product produced in 2×10^{-3} M H_2O_2 (less efficient), and (d) reduction product obtained in 2×10^{-3} M $N_2H_6SO_4$ (most destructive).

Mesoporous silver film was prepared in two steps using a magnetron sputtered silver films of 150 nm thick as a precursor. The primary Ag film was deposited onto thoroughly washed glass slide in argon using magnetron Quorum Technologies Q150T Turbo-Pumped Sputter Coater/Carbon Coater. A standard silver disk sputtering target (Stanford Materials, 99.99%) served as a source and the deposition rate was 2 nm/s, current 50 mA. The film was even and had less defects.

The silver film was oxidized then in vapors of concentric nitric acid (Reachim, purum). The silver film was fixed downface in 5 cm over 10 mL of acid in a glass vessel. The film was treated for 10 min in vapor and then thoroughly washed with MilliQ pure water. The film changed in color was then immersed into fresh prepared $NaBH_4$ aqueous solutions of the same concentration as was taken for mesoporous polyhedrons when taken in ten-fold excess. The initial concentration of $NaBH_4$ was of 1×10^{-2} M. The immersion time was 15 min and 40 min to vary process rate. To reveal the influence of PVP onto morphology of the metal silver one of

reductant solutions contained also 5×10^{-3} M of PVP. The resulting plates were washed with distilled water and dried in ambient conditions for 24 h.

Scanning electron microscopy (SEM) was applied to characterize microstructure of the individual Ag and/or Ag_2O nanostructures. The analysis has been performed in using NVision 40 microscope (Carl Zeiss) at 9 kV accelerating voltage. The instrument was equipped with an Oxford Instruments X-Max detector. Statistical analysis of micrographs was performed using ImageJ software.

XRD spectra were collected using the X-ray diffractometer RIGAKU D/max-RC with 12 kW beam gun and a rotating copper anode (Cu Kα radiation, θ−2θ Bragg−Brentano geometry, 20−60° 2θ range, 0.020° step). Phase analysis of compounds was carried out using WinXPow software using PDF2 database.

X-ray photoelectron spectroscopy analysis was performed using a setup ESCA I at the Laboratory of Industrial Chemistry of RUB equipped with the X-ray source Specs XRC 1000, UHV chamber up to 10^{-6} mbar, energy analyzer power PS-EA10N). For the sample preparation the preliminary centrifuge concentrated colloid was deposited onto the carbon substrate and then evaporated for 10 times.

Specific surface area of samples was determined by low-temperature nitrogen adsorption with an ATX-06 analyzer (KATAKON) by the five-point Brunauer–Emmett–Teller (BET) method. The characteristic nitrogen adsorption–desorption isotherms are given in Figure S2.

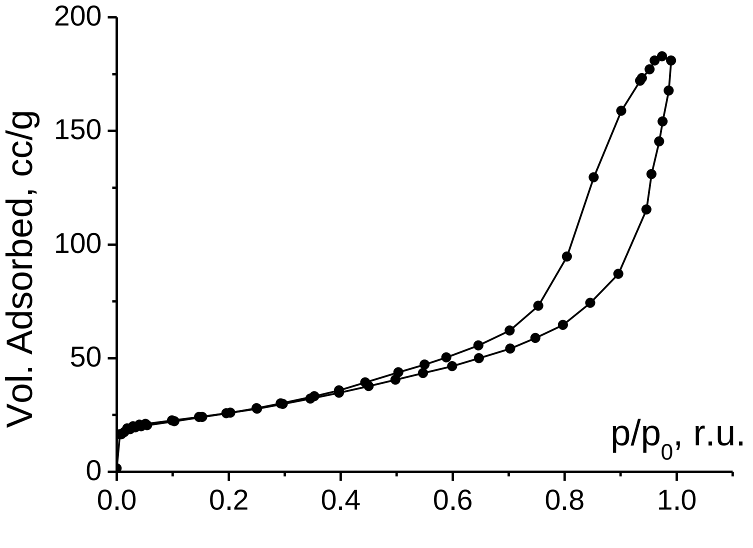

Figure S2: Nitrogen adsorption plot for the Ag mesoporous nanocubes sample synthesized with the molar ratio of compounds of 1 Ag_2O to 10 $NaBH_4$.

All SERS spectra have been collected using an InVia Raman confocal microscope (Renishaw Inc.) equipped with a 20 mW 514.4 nm argon laser and power neutral density filter (10%) with 60 s of acquisition time. All spectra have been collected using a confocal microscope Leica DMLM (resolution up to 2.5 μm) with 50× objective lens. Diffraction grating was 2400 lines/mm, CCD camera 1024 × 256 pixels. A standard (100) single crystalline silicon substrate has been used for calibration. As a reference sample of rhodamine 6G solution deposited onto inert silica substrates in 10^{-3} M concentration was applied. The reference raman spectra of mildronate was obtained using the 100 g/mL solution deposited onto a microbiological glass substrate. The aliquote volume was of ~1–2 μL in all Raman measurements performed.

The water wetting contact angles of nanostructured films were measured on a Leica optical micro scope with a magnification 50×. The experimental sample was placed on a horizontal holder. In order to avoid the measurement errors caused by the gravity induced distortion of the shape of a drop, each drop was deposited onto the surface by means of a special microdose syringe. The volume of the drop was 1 μL.

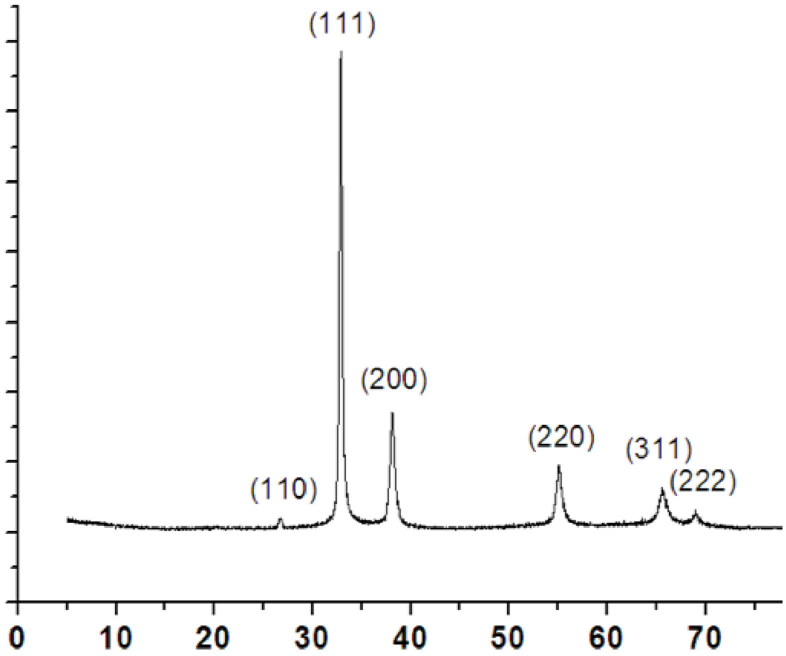

(a)

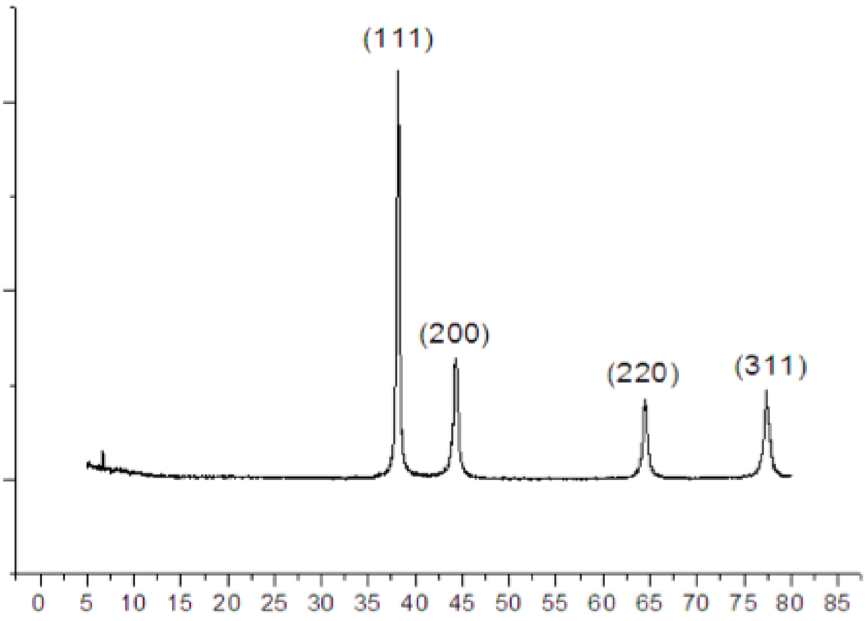

(b)

Figure S3: XRD data for Ag mesoporous films synthesized with the molar ratio of compounds of 1 Ag_2O to 10 $NaBH_4$ in (a) 15 min and (b) 40 min reduction process.

In Figure S4 the micrographs of mp-Ag/Ag film are presented. The film was not delaminated successfully from the glass slide for additional SEM experiment (because it was too thin to keep its planar structure). The current micrographs show uniform micromorphology for two sides of the film neat the edge which formed a gather.

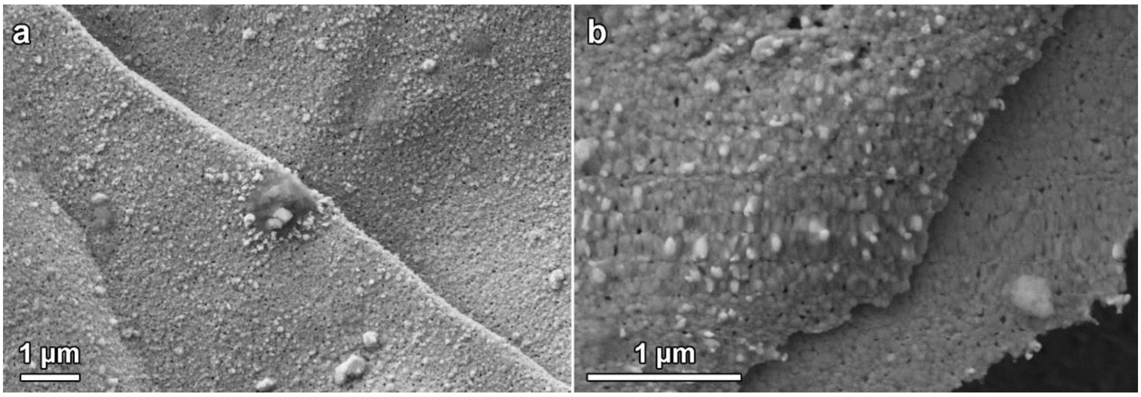

Figure S4: SEM data for Ag mesoporous films (mp-Ag/Ag) synthesized with the molar ratio of compounds of 1 Ag_2O to 10 $NaBH_4$ with 40 min reduction process in presence of 5 mM PVP.

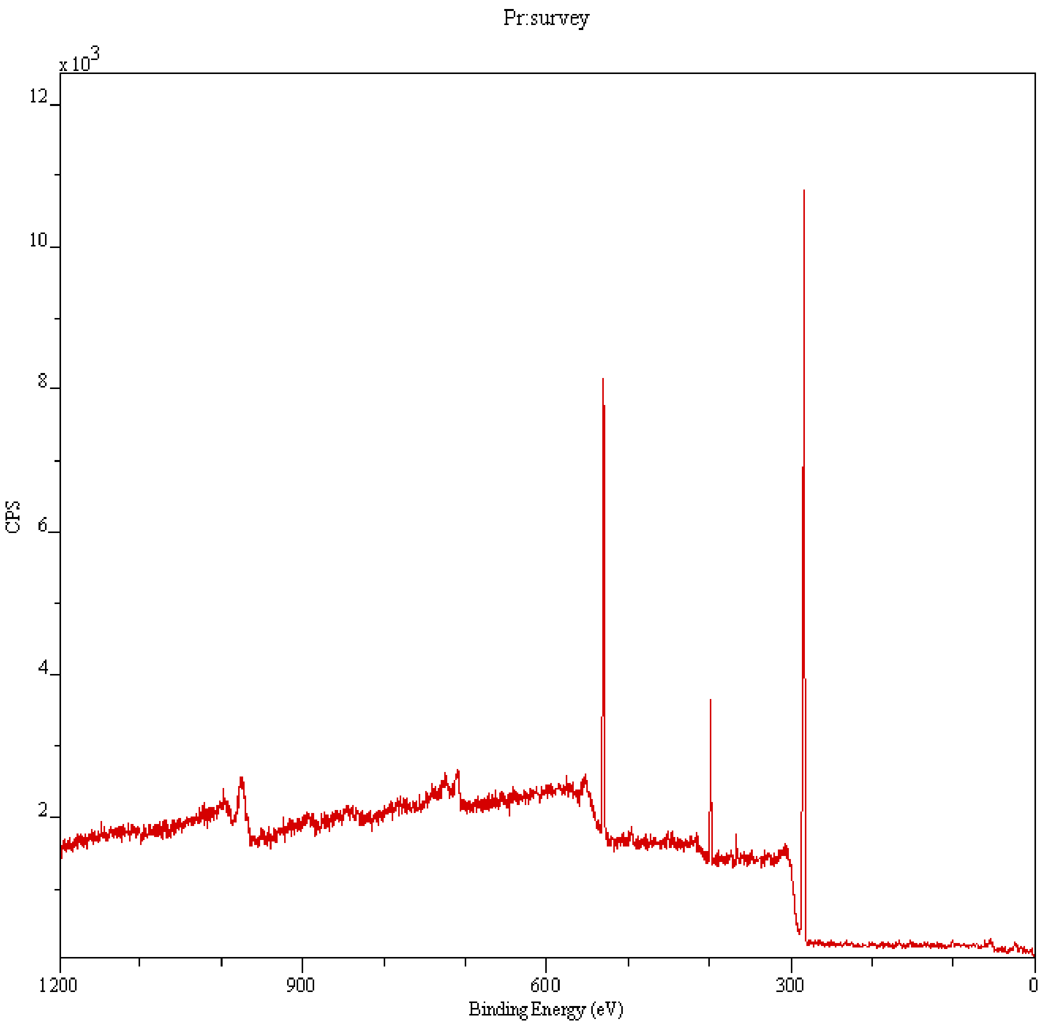

Figure S5: The XPS data for Ag mesoporous aggregates obtained of 1 crystalline Ag_2O to 10 $NaBH_4$ with 40 min of reduction process. The suspension was 10 times dropped onto a conducting sample holder to reach an appropriate signal values for Ag.

References

1. Lyu, L.-M.; Wang, W.-Ch.; Huang, M. H. *Chem. – Eur. J.* **2010,** *16*, 14167–14174. doi:10.1002/chem.201000563
